# A review of the Zoogonidae (Digenea: Microphalloidea) from fishes of the waters around New Caledonia, with the description of *Overstreetia cribbi* n. sp.

**DOI:** 10.7717/peerj.292

**Published:** 2014-03-13

**Authors:** Rodney A. Bray, Jean-Lou Justine

**Affiliations:** 1 Department of Life Sciences, Natural History Museum, London, UK; 2 ISYEB, Institut de Systématique, Évolution, Biodiversité, Muséum National d’Histoire Naturelle, France

**Keywords:** Zoogonidae, New Caledonia, *Atherinomorus*, Fish parasite, Digenea, *Overstreetia cribbi* n. sp.

## Abstract

New and published reports of zoogonid digeneans from New Caledonian waters are recorded, including a description of *Overstreetia cribbi* n. sp. from *Atherinomorus lacunosus*. This species differs from its congeners in the detail of its circum-oral spination and some metrical features. Other new records are of: *Diphterostomum plectorhynchi* Machida, Kamegai & Kuramochi, 2006 in *Diagramma pictum*; *Parvipyrum acanthuri* (Pritchard, 1963) in *Acanthurus dussumieri*; *Zoogonoides viviparus* (Olsson, 1868) in *Lagocephalus sceleratus*; *Deretrema ? combesorum* (Bray & Justine, 2008a; Bray & Justine, 2008b) early ovigerous forms in *Parupeneus pleurostigma*; *D*? *acutum* (Pritchard, 1963) in *P. barberinus*; and an unidentified immature zoogonid in *P. multifasciatus*. The newly reported specimens are illustrated and measurements given. The distribution of New Caledonian zoogonids is listed.

## Introduction

Early systematic studies of the marine digenean fauna of New Caledonia were by Durio & Manter (1968a), Durio & Manter (1968b) and Durio & Manter (1969). More recently we have added to the knowledge of this fauna, including summary papers on the Lepocreadiidae Odhner, 1905 (Bray & Justine, 2012) and the Bucephalidae Poche, 1907 (Bray & Justine, 2013). Here we present a similar paper summarising our knowledge of the family Zoogonidae Odhner, 1902. This family is one of the major families of marine fish digeneans, with a few species in freshwater (Bray, 2008). It contains 159 species in 33 genera. Molecular analyses of a few species (Hall, Cribb & Barker, 1999; Cribb et al., 2001; Olson et al., 2003; Bray et al., 2005; new data) shows that the family is a member of the superfamily Microphalloidea Ward, 1901, close to, and probably paraphyletic to the family Faustulidae Poche, 1926. There are two distinct subfamilies in the Zoogonidae, the Zoogoninae Odhner, 1902, whose members produce eggs without a tanned shell, and therefore have a reduced vitellarium (Bray, 1987a), and the Lepidophyllinae Stossich, 1903, whose members produce tanned eggs and have a vitellarium consisting of paired fields of follicles (Bray, 1987b). Molecular phylogeny (Olson et al., 2003; Bray et al., 2005) indicates that the Zoogoninae, based on two species, is monophyletic and sister to the monophyletic Faustulidae, based on three species. The two species of lepidophyllines in the analysis are paraphyletic, with the *Deretrema* species sister to the Zoogoninae + Faustulidae, and *Lepidophyllum* species sister to that assemblage. As can be seen the sample size is small and, although the support for this arrangement is statistically good, the findings are clearly provisional and preliminary. The known zoogonid fauna of waters around New Caledonia is small, but will probably be found to be larger when explorations of the deep-sea are undertaken. In this paper we list all the known species, including a new species of the genus *Overstreetia* (Bray, 1985), and attempt to place them in the context of their endemicity.

## Materials and Methods

Digeneans were collected live, immediately fixed in nearly boiling saline (Cribb & Bray, 2010; Justine, Briand & Bray, 2012) and then transferred to 80% ethanol. Whole-mounts were stained with Mayer’s paracarmine, cleared in beechwood creosote and mounted in Canada balsam. Measurements were made through a drawing tube on an Olympus BH-2 microscope, using a Digicad Plus digitising tablet and Carl Zeiss KS100 software adapted by Imaging Associates, and are quoted in micrometres. The following abbreviations are used: BMNH, British Museum (Natural History) Collection at the Natural History Museum, London, UK; MNHN JNC, Muséum National d’Histoire Naturelle, Paris, France.

The electronic version of this article in Portable Document Format (PDF) will represent a published work according to the International Commission on Zoological Nomenclature (ICZN), and hence the new names contained in the electronic version are effectively published under that Code from the electronic edition alone. This published work and the nomenclatural acts it contains have been registered in ZooBank, the online registration system for the ICZN. The ZooBank LSIDs (Life Science Identifiers) can be resolved and the associated information viewed through any standard web browser by appending the LSID to the prefix “http://zoobank.org/”. The LSID for this publication is: urn:lsid:zoobank.org:act:FC32CC29-F6DD-46DC-B2F2-84F3953B2991. The online version of this work is archived and available from the following digital repositories: PeerJ, PubMed Central and CLOCKSS.

**Family Zoogonidae Odhner, 1902**

**Subfamily Zoogoninae Odhner, 1902**

**Genus** ***Diphterostomum*** **Stossich, 1903**

ZooBank: urn:lsid:zoobank.org:act:D56035F2-141C-4607-8AFE-7AEFED0AE0B3.

***Diphterostomum tropicum*** **Durio & Manter, 1968**

ZooBank: urn:lsid:zoobank.org:act:71827EC5-F9EA-44A4-888D-12BF3E1FE770.

Syn: *Diphtherostomum tropicum* Durio & Manter, 1968

Record from off New Caledonia: Durio & Manter (1968a)

New Caledonian host: as *Lethrinus* sp., ‘bec de cane’; ‘can be safely identified as’ *Lethrinus nebulosus* (Forsskål, 1775) (Justine, 2007).

Discussion: Durio & Manter (1968a) also reported this species in the pink-ear emperor *Lethrinus lentjan* (Lacepède, 1802) (as *L. glyphodon* Günther, 1859) from Green Island, on the Great Barrier Reef. The only other report is from the sparid *Chrysophrys auratus* (Forster, 1801) from off New Zealand by Korotaeva (1975).

**Figure 1:**
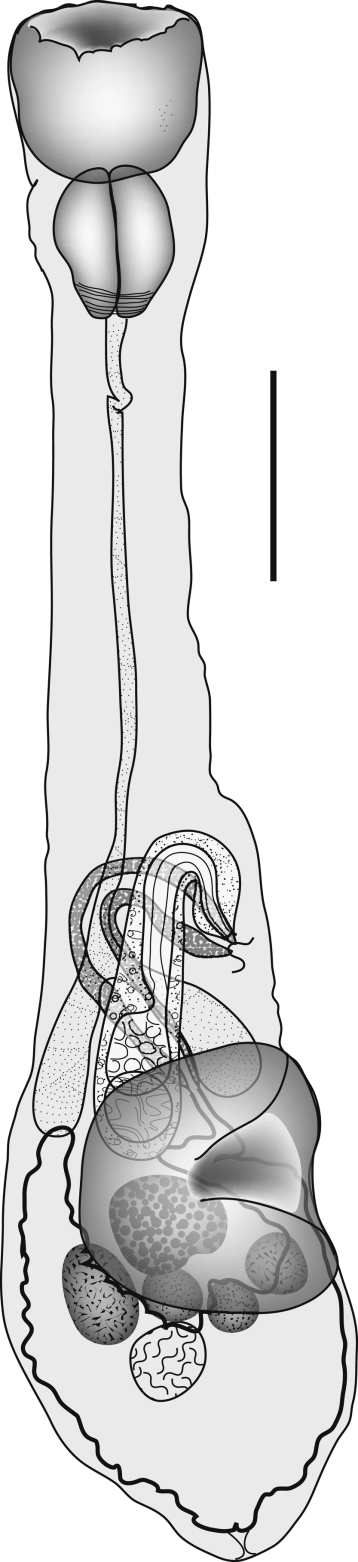
*Diphterostomum plectorhynchi.* *Diphterostomum plectorhynchi* Machida, Kamegai & Kuramochi 2006. Ventral view with ventral sucker twisted. Scale bar 200 µm.

***Diphterostomum plectorhynchi*** **Machida, Kamegai & Kuramochi, 2006** (Fig. 1)

ZooBank: urn:lsid:zoobank.org:act:3949FC49-247A-49CB-85E3-208E7FF8551C.

Host: *Diagramma pictum* (Thunberg, 1792), Perciformes, Haemulidae, painted sweetlips.

Site: digestive tract.

Locality: Between Larégnière and Récif Crouy (22°20′702S, 166°19′295E, 05/05/2008); Interior Lagoon near Récif Toombo (22°32′536S, 166°29′069E, 25/08/2009).

Specimens: MNHN JNC2511, JNC2512, JNC3023, BMNH 2014.1.31.2-3.

Previous New Caledonian records: none.

Discussion: This species was originally reported from this host and two *Plectorhinchus* spp. from off Japan (Machida, Kamegai & Kuramochi, 2006). The specimens originally described were flattened at fixation, so some features appear distinct, but the major distinguishing feature of this species is the large ‘elongate conical’ pharynx. Taking into consideration the different fixation methods used, our specimens do not appear to be distinguishable from *D. plectorhinchi* (Table 1).

**Table 1:** Measurements and ratios of three zoogonine species.

Species	*Diphterostomum plectorhynchi*			*Parvipyrum acanthuri*		*Zoogonoides viviparus*
Host	*Diagramma pictum*			*Acanthurus dussumieri*		*Lagocephalus sceleratus*
*n*	10			2		1
	min	max	mean			
Length	880	1,395	1,120	511	592	561
Width	213	333	275	Lateral	Lateral	233
Forebody length	545	929	699	183	150	219
Oral sucker length	62	212	147	68	58	97
Oral sucker width	89	198	151	Lateral	Lateral	92
Prepharynx length	0	0	0	11	10	0
Pharynx length	53	198	113	28	28	35
Pharynx width	38	121	85	Lateral	Lateral	43
Oesophagus length	295	508	416	81	77	50
Intestinal bifurcation to ventral sucker	18	82	62	0	0	53
Pre-vitelline distance	729	1,059	860	?	404	382
Vitelline mass length	40	74	56	?	31	43
Vitelline mass width	42	61	53	?	32	44
Ventral sucker length	153	269	223	224	279	155
Ventral sucker width	159	209	186	Lateral	Lateral	175
Cirrus-sac length	211	424	320	159	187	97
Cirrus-sac width	59	78	67	51	50	43
Ovary length	78	112	95	93	103	105
Ovary width	75	143	106	76	70	61
Testis length	61	128	97	80	91	?
Testis width	52	120	79	81	77	?
Post-testicular distance	75	171	136	77	78	?
Post-vitelline distance	83	219	165	112	136	140
Post-uterine distance	14	69	34	25	20	21
Post caecal distance	227	449	361	246	291	196
Egg length	31	41	36	29	46	42
Egg width	11	22	15	19	18	23
Width %^*^	20.9	28.1	24.5	Lateral	Lateral	41.5
Forebody %^*^	57.6	66.6	62.3	35.8	25.3	39.0
Sucker length ratio	1.06	2.88	1.65	3.31	4.84	1.59
Sucker width ratio	0.95	1.94	1.39	Lateral	Lateral	1.90
Pharynx: oral sucker width ratio	0.43	0.65	0.55	Lateral	Lateral	0.46
Oral sucker length %^*^	6.10	15.9	13.0	13.2	9.7	17.4
Pharynx length %^*^	5.46	14.2	9.88	5.53	4.80	6.17
Ventral sucker length %^*^	16.1	24.7	20.0	43.8	47.1	27.7
Oesophagus length %^*^	24.7	48.4	35.8	15.9	13.0	8.92
Pre-vitelline distance %^*^	76.5	84.4	80.0	?	68.24	68.1
Ovary length %^*^	6.55	9.80	8.56	18.2	17.4	7.70
Testis length %^*^	6.74	10.8	8.71	15.6	15.4	18.7
Post-testicular distance %^*^	8.32	13.7	11.8	15.1	13.2	?
Post-vitelline distance %^*^	9.27	18.5	14.5	21.8	22.9	24.9
Post-uterine distance %^*^	1.35	5.76	3.06	4.90	3.45	3.67
Postcaecal distance %^*^	18.7	35.6	29.0	24.1	24.6	36.9
Cirrus-sac length %^*^	17.6	41.7	29.4	31.1	31.6	17.3
Intestinal bifurcation to ventral sucker %^**^	3.18	10.36	7.96	?	?	24.1

**Notes.**

* % of body-length.

** % of forebody.

Two other species of *Diphterostomum* have been reported in haemulid fishes, *D. anisotremi* (Nahhas & Cable, 1964) and *D. indicum* (Madhavi, 1980). The former is an Atlantic species with a tiny pharynx and is easily distinguishable from *D. plectorhynchi* (see Nahhas & Cable, 1964) and has been considered synonymous with *D. brusinae* (Stossich, 1888) (Bray, 1987a). *D. indicum*, reported from the silver grunt *Pomadasys argenteus* (Forsskål, 1775) (as *P. hasta*), the banded grunter *P. furcatus* (Bloch & Schneider, 1801) (as *Rhonciscus furcatus*) and the saddle grunt *P. maculatus* (Bloch, 1793) from the Bay of Bengal (Madhavi, 1980), is said to have both dextral and sinistral genital pores, and has a fairly small pharynx. This seems the most similar species to *D. plectorhynchi*.

There are about 13 recognised *Diphterostomum* spp. (depending on the validity of some synonymies) with, according to our records, over 200 reports of various life-cycle stages. More than 130 of these (66%) refer to the species *D. brusinae*. About 5% of these records are from the Indo-West Pacific Region, but no reports are from haemulids. Members of this genus are all similar with few distinguishing features.

**Genus** ***Parvipyrum*** **Pritchard, 1963**

ZooBank: urn:lsid:zoobank.org:act:7C176519-62E1-401E-BDDE-F19F8B2CBA7D.

**Figure 2:**
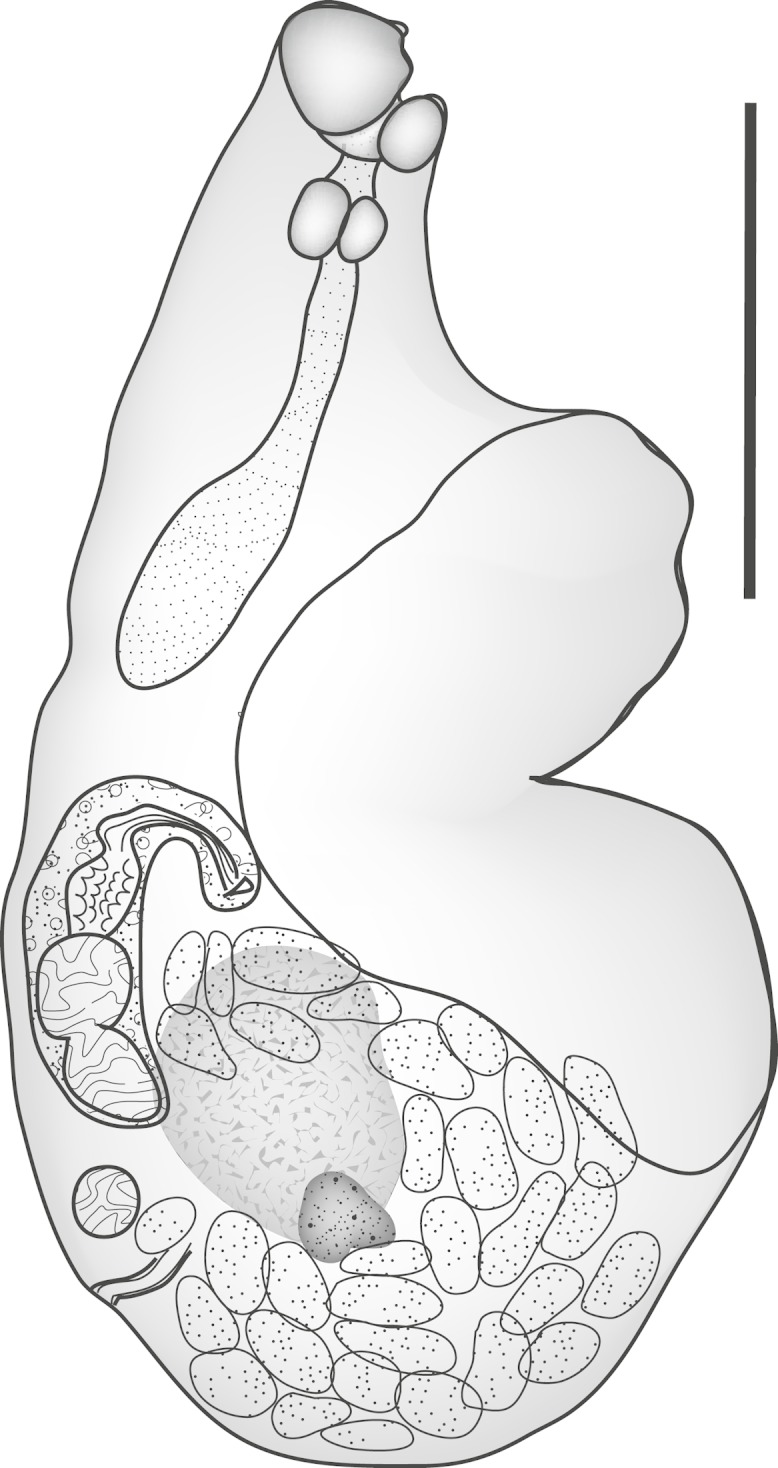
*Parvipyrum acanthuri.* *Parvipyrum acanthuri* Pritchard, 1963. Lateral view. Scale bar 200 µm.

***Parvipyrum acanthuri*** **Pritchard, 1963** (Fig. 2)

ZooBank: urn:lsid:zoobank.org:act:CC2194F2-9241-43D4-9E17-0E5EFCA3C9A3.

Hosts: *Acanthurus blochii* Valenciennes, 1835, Perciformes, Acanthuridae, ringtail surgeonfish; *A. dussumieri* Valenciennes, 1835, Perciformes, Acanthuridae, eyestripe surgeonfish.

Site: Digestive tract.

Localities: ex *A. blochii*, Nouméa Fish Market (23/06/2007); ex *A. dussumieri*, Nouméa Fish Market (08/04/2011), Récif Snark (22°26′S, 166°25E, 15/05/2008).

Specimens: ex *A. blochii*, MNHN JNC2213, BMNH 2007.11.14.51; ex *A. dussumieri*, MNHN JNC2545, JNC3374, BMNH 2014.1.31.6.

Previous New Caledonian record: Bray & Justine (2008a).

Previously reported New Caledonian host: *Acanthurus blochii*.

Discussion: This tiny worm (Table 1) is known only from members of the genus *Acanthurus*, and has been reported only from Hawaii (Pritchard, 1963; Yamaguti, 1970) and New Caledonia.

**Genus** ***Zoogonoides*** **Odhner, 1902**

ZooBank: urn:lsid:zoobank.org:act:6CEFCB5C-4537-4C5D-A1F7-08901790BF65.

**Figure 3:**
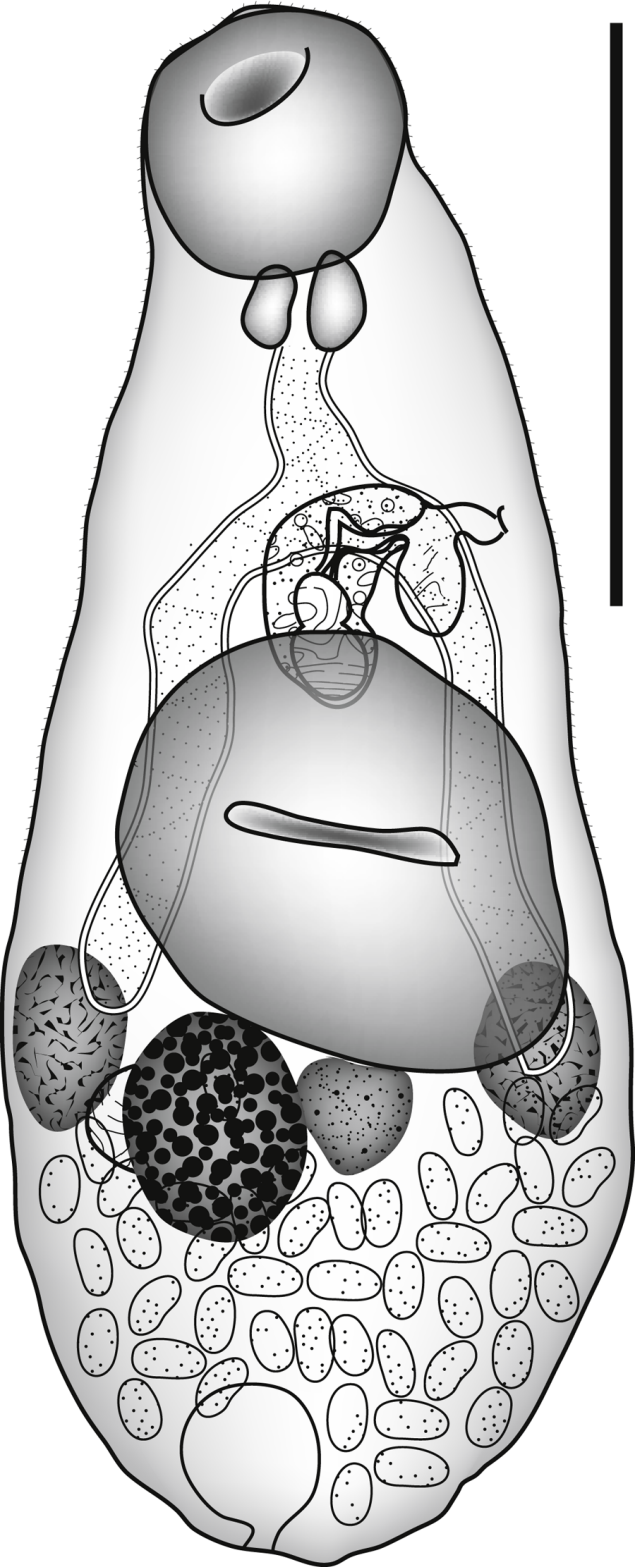
*Zoogonoides viviparus.* *Zoogonoides viviparus* (Olsson, 1868). Ventral view. Scale bar 200 µm.

***Zoogonoides viviparus*** **(Olsson, 1868) Odhner, 1902** (Fig. 3)

ZooBank: urn:lsid:zoobank.org:act:A1FAB647-C92F-4E1B-8528-333DA6931E3E.

Synonyms: see Bray & Gibson (1986).

Host: *Lagocephalus sceleratus* (Gmelin, 1789), Tetraodontiformes, Tetraodontidae, silver-cheeked toadfish.

Site: digestive tract.

Locality: near Îlot Pandanus (22°15′585S, 166°17′513E, 18/06/2009).

Specimens: MNHN JNC2982.

Previous New Caledonian records: none.

Discussion: Seven nominal species of *Zoogonoides* have been described from the Indo-Pacific region. Three of these can be distinguished from our specimen by the sucker ratio, with the ventral sucker smaller than, or of similar size to, the oral sucker (Table 1): *Z. acanthogobii* Yamaguti, 1938, *Z. kamegaii* Toman, 1992 and *Z. anampses* Toman, 1992; (Yamaguti, 1938; Toman, 1992). Two others, *Z. pyriformis* Pritchard, 1963 and *Z. synodi* Yamaguti, 1970; (Pritchard, 1963; Yamaguti, 1970) can be distinguished by the lack of an atrial sac, a feature which is clear in the single specimen we have (Fig. 3). The two Indo-Pacific species that are described with this feature are the type-species, *Z. viviparus* (Olsson, 1868), and *Z. yamagutii* Kamegai, 1973. *Z. viviparus* is reported mainly in the North Atlantic Ocean, having been originally reported off Norway (Olsson, 1868; Bray & Gibson, 1986), but has been reported in the northern Pacific Ocean (Zhukov, 1960; Mamaev, Parukhin & Baeva, 1963; Machida et al., 1972; Tsimbalyuk, 1978; Machida, 1984; Shimazu, 1984) and the northern Indian Ocean (Sujatha & Madhavi, 1990). *Z. yamagutii* is known only from *Plotosus lineatus* (Thunberg, 1787) [as *P. anguillaris*] (Siluriformes: Plotosidae) from Nishidomari Bay, Tsushima Island, Japan (Kamegai, 1973). It was originally (Kamegai, 1973) compared only with *Z. acanthogobii* which was considered the ‘only other representative in the genus possessing a saccular posterior diverticle of the genital atrium’. In fact, *Z. viviparus* is now known to exhibit this feature (see Bray & Gibson, 1986) and we cannot see any distinction between this species and our specimen. As far as we are aware this is the first record of *Zoogonoides* from the Southern Hemisphere apart, possibly, from the dubious report of *Zoogonoides* sp. from a freshwater fish in Lake Victoria, Uganda (Akoll et al., 2012). Aspects of the life-cycle of *Z. viviparus* are known from the northeastern Atlantic. The first intermediate host is the sorbeoconchan gastropod *Buccinum undatum* (Linnaeus, 1758), the second in intermediate host may be an ophiuroid or holothurian echinoderm, a polychaete, bivalve or gastropod, or possibly a mysid crustacean (Køie, 1976; Bray & Gibson, 1986). The most frequently reported definitive hosts are flatfish, with about 75% of records (pleuronectids 68%, soleids 6.6%, scophthalmids 0.9%). The only other group commonly reported as hosts are the callionymids (8.5%) and there are occasional reports from anarhichadids, blenniids, gobiids, liparids, lophiids, sillaginids, sticheaids and zeids, with one report from a cyprinid. Therefore, this appears to be the first report of *Zoogonoides* in a tetraodontiform fish. It is likely that *Z. viviparus* is a complex of species awaiting molecular elucidation.

**Genus** ***Zoogonus*** **Looss, 1901**

ZooBank: urn:lsid:zoobank.org:act:A7BE318D-98F2-49C3-975E-5ED3D691ACEF.

***Zoogonus pagrosomi*** **Yamaguti, 1939**

ZooBank: urn:lsid:zoobank.org:act:FE7F7E55-FD83-4021-A271-A9B5BA66F1DF.

Hosts: *Gymnocranius euanus* (Günther, 1879), Perciformes, Lethrinidae, Japanese large-eye bream; *Lethrinus atkinsoni* Seale, 1910, Perciformes, Lethrinidae, Pacific yellowtail emperor; *L. genivittatus* Valenciennes, 1830, Perciformes, Lethrinidae, longspine emperor:

Site: intestine, digestive tract.

Localities: ex *G. euanus*, Inside Lagoon, facing Récif Toombo (22°32′361S, 166°26′992E, 06/11/2007), Off Récif Kué (22°36′S, 166°31′E, 07/10/2008); ex *L. atkinsoni*, Off Ever Prosperity (22°27′S, 166°21′E, 26/04/2006); ex *L. genivittatus*, Off Baie des Citrons, Nouméa (22°17′55″S, 166°25′20″E, 21/07/2007), Baie Maa (22°12′809S, 166°19′666E, 30/08/2007).

Specimens: ex *G. euanus*, MNHN JNC2388, BMNH 2014.1.31.4; ex *L. atkinsoni*, MNHN JNC1789, BMNH 2007.11.14.52; ex *L. genivittatus*, MNHN JNC2293, BMNH 2007.11.14.52.

Previous New Caledonian records: 1. Bray & Justine (2008a), 2. Justine et al. (2010).

Previously reported New Caledonian host: *G. euanus* (2, as *Zoogonus* sp.), *L. atkinsoni* (1, 2), *L. genivittatus* (1, 2).

Discussion: We have recovered this species only from lethrinid fishes, but it was originally described from the sparid *Chrysophrys auratus* Forster 1801 (as *Pagrosomus unicolor*) from the Inland Sea of Japan (Yamaguti, 1939). Cribb, Bray & Barker (1992) reported it in *Lethrinus atkinsoni* off Heron Island in the southern Great Barrier Reef. The only other record of which we are aware is from the gadiform *Merluccius gayi peruanus* Ginsburg, 1954 (Merlucciidae) from off Callao, Peru (Rivera Terrones, 1992). This appears to be a poorly known species, therefore, with an unusual distribution, both geographical and in terms of its hosts.

**Subfamily Lepidophyllinae Stossich, 1903**

**Genus** ***Deretrema*** **Linton, 1910**

ZooBank: urn:lsid:zoobank.org:act:17B66089-6C3B-4FF8-A89A-526FBC4C00E0.

***Deretrema combesae*** **Bray & Justine, 2008**

ZooBank: urn:lsid:zoobank.org:act:9BAE2C81-2145-4704-9AD9-E1FFEAD06603.

Record from off New Caledonia: Bray & Justine (2008b).

New Caledonian host: *Parupeneus multifasciatus* (Quoy & Gaimard, 1825), Perciformes, Mullidae, manybar goatfish.

***Deretrema combesorum*** **Bray & Justine, 2008**

ZooBank: urn:lsid:zoobank.org:act:CE93F29C-4B9E-4E39-B986-BBB772DA8016.

Record from off New Caledonia: Bray & Justine (2008b).

New Caledonian host: *Parupeneus multifasciatus* (Quoy & Gaimard 1825), Perciformes, Mullidae, manybar goatfish.

**Figure 4:**
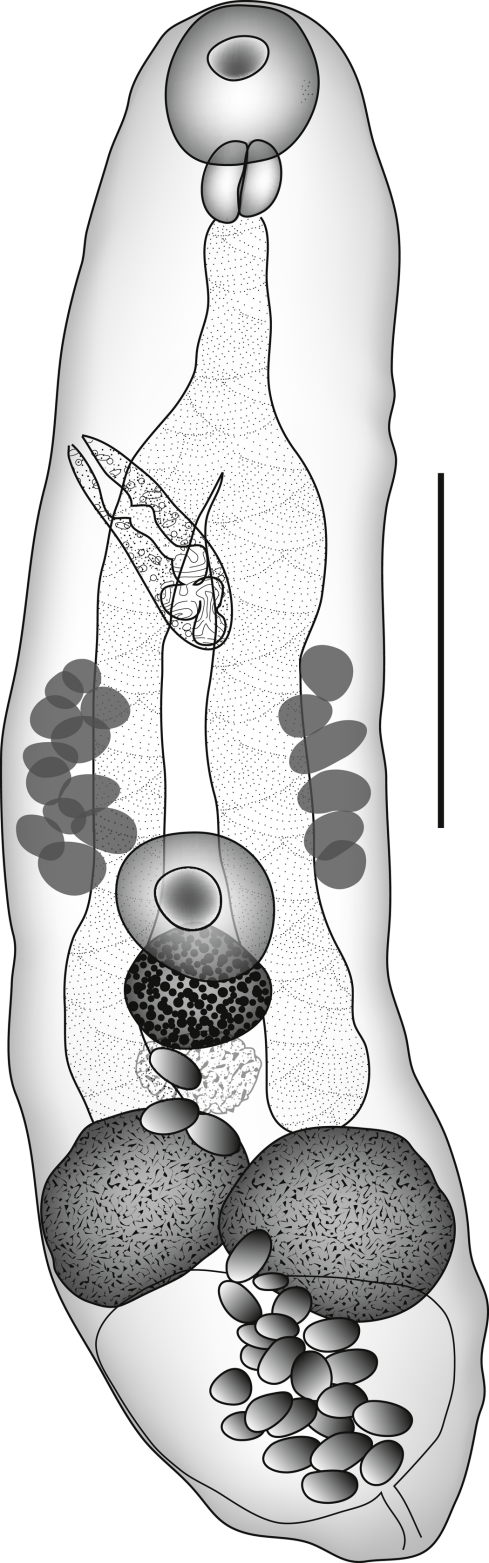
*Deretrema ? combesorum.* *Deretrema ? combesorum* Bray & Justine, 2008, early ovigerous form. Scale bar 200 µm.

***Deretrema ? combesorum*** **Bray & Justine, 2008, early ovigerous forms** (Fig. 4)

Host: *Parupeneus pleurostigma* (Bennett, 1831), Perciformes, Mullidae, sidespot goatfish.

Site: digestive tract

Locality: West of Passe de Dumbéa (precise coordinates not available, 22/11/2007).

Specimens: MNHN JNC2416, BMNH 2014.1.31.5.

Discussion: Three small worms, two of them with a few eggs, may be early mature members of *C. combesorum*. They show all the diagnostic characters listed in Bray & Justine (2008b, table 2), differentiating *D. combesae* and *C. combesorum*. Nevertheless, several metric and ratio characters differ, e.g., size, width ratio, and the ratios of the suckers and the sizes of most gonads relative to body-length (Table 2). These ratios may represent allometric growth, or they may indicate that this is a distinct, but similar species. The information we have to hand is not sufficient to decide between these alternatives and is certainly not enough to warrant the erection of a new species.

**Table 2:** Measurements and ratios of *Deretrema* spp.

Species	*Deretrema* ? *acutum*	*Deretrema* ? *combesorum*		
Host	*Parupeneus barberinus*	*Parupeneus pleurostigma*		
*n*	1	min	max	mean
Length	2,035	721	935	794
Width	487	214	278	245
Forebody length	674	351	489	412
Oral sucker length	203	78	96	89
Oral sucker width	206	85	91	88
Prepharynx length	7	0	0	0
Pharynx length	101	47	53	50
Pharynx width	91	45	50	47
Oesophagus length	173	71	113	93
Intestinal bifurcation to ventral sucker	180	154	246	195
Pre-vitelline distance	560	303	382	333
Vitelline field length	363	120	148	136
Ventral sucker length	298	83	92	88
Ventral sucker width	375	90	94	93
Cirrus-sac length	345	112	145	127
Cirrus-sac width	69	39	50	46
Ovary length	161	53	104	86
Ovary width	149	74	92	85
Testis length	191–219	96	112	106
Testis width	142–166	83	141	111
Post-testicular distance	789	61	144	101
Post-vitelline distance	1,098	277	393	323
Post-uterine distance	114	40	44	42
Post caecal distance	777	175	249	204
Egg length	42	28	41	34
Egg width	22	14	23	19
Width %^*^	23.94	**25.9**	**38.5**	**31.3**
Forebody %^*^	33.13	**48.7**	**54.5**	**51.9**
Sucker length ratio	1.47	0.89	1.18	1.00
Sucker width ratio	1.81	1.03	1.06	1.05
Pharynx: oral sucker width ratio	0.44	0.50	0.56	0.53
Oral sucker length %^*^	9.97	**10.26**	**12.95**	**11.30**
Pharynx length %^*^	4.96	5.27	7.31	6.35
Ventral sucker length %^*^	14.7	9.59	12.66	11.24
Oesophagus length %^*^	8.52	9.79	13.29	11.73
Pre-vitelline distance %^*^	27.5	40.90	43.27	42.09
Vitelline field length%^*^	17.8	15.86	19.15	17.20
Ovary length %^*^	7.89	7.38	14.36	10.79
Testis length %^*^	9.40	11.96	14.65	13.65
Post-testicular distance %^*^	38.8	8.33	15.39	12.47
Post-vitelline distance %^*^	54.0	38.06	42.06	40.59
Post-uterine distance %^*^	5.62	4.69	5.48	5.09
Postcaecal distance %^*^	34.7	22.94	27.72	25.33
Cirrus-sac length %^*^	17.0	15.46	17.26	16.09
Intestinal bifurcation to ventral sucker %^**^	26.7	43.75	50.24	46.84

**Notes.**

* % of body-length.

** % of forebody.

*Deretrema triodontis* **Machida & Kuramochi, 1999**

ZooBank: urn:lsid:zoobank.org:act:58F09856-2B9D-45E3-90A1-9501B76CC397

Record from off New Caledonia: Bray, Cribb & Justine (2010).

New Caledonian host: *Triodon macropterus* Lesson, 1831, Tetraodontidae, Triodontidae, threetooth puffer.

Discussion: This species was originally reported in this host, from Okinawa, Japan (Machida & Kuramochi, 1999). Our record from New Caledonia is the only other report of this species.

**Figure 5:**
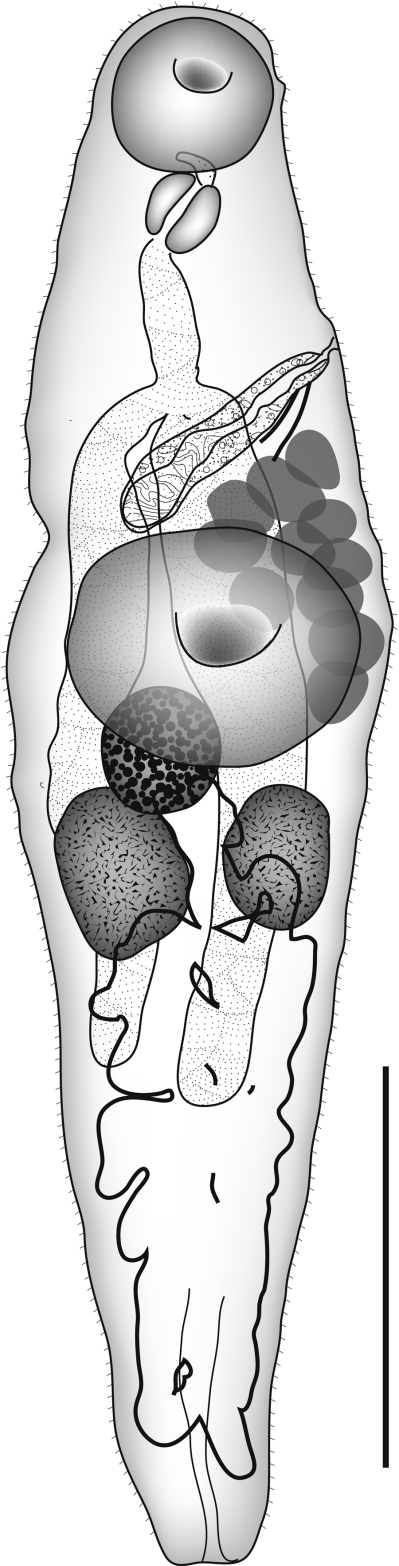
*Deretrema*? *acutum.* *Deretrema*? *acutum* Pritchard, 1963. Ventral view. Scale bar 500 µm.

***Deretrema ? acutum** Pritchard, 1963* (Fig. 5)

ZooBank: urn:lsid:zoobank.org:act:CC7B648A-0BF1-400C-BFF6-B4852742B4E9.

Host: *Parupeneus barberinus* (Lacepède, 1801), Perciformes, Mullidae, dash-and-dot goatfish.

Site: digestive tract.

Locality: West of Îlot Goëland (22°22′246S, 166°22′934E, 26/10/2007).

Specimens: MNHN JNC2346.

Previous New Caledonian records: none.

Discussion: The single specimen available appears indistinguishable (Table 2) from *D. acutum* as described by Pritchard (1963) apart from the vitellarium which is developed on one side of the body only. As only one specimen is available it is not possible to be certain that this is an anomalous condition. Both previous reports of this species, under this name, are from acanthurids of the genus *Naso*, from off Hawaii (Pritchard, 1963; Yamaguti, 1970). Bray (1987b) considered *D. hawaiiense* Yamaguti, 1970, *D. sphyraenae* Yamaguti, 1970, *D. uku* Yamaguti, 1970 and *Deretrema* sp. of Yamaguti (1951) synonymous with *D. acutum*, following the discussion of Beverley-Burton & Early (1982) who pointed out that, according to Yamaguti (1970), they are distinguished by minor details of Laurer’s canal. If these synonymies are accepted, then the host list is increased to include cheilodactylids, carangids, lutjanids and sphyraenids, and the distribution is widened to include Japanese waters.

**Genus** ***Dupliciporia*** **Reimer, 1985**

ZooBank: urn:lsid:zoobank.org:act:78C451F6-4544-4FB1-89F4-95CD079874FF.

***Dupliciporia lanterna*** **Bray & Justine, 2008**

ZooBank: urn:lsid:zoobank.org:act:29BD2E10-7E06-4F86-90EA-C8840F1A5432.

Record from off New Caledonia: Bray & Justine (2008a).

New Caledonian host: *Priacanthus hamrur* (Forsskål, 1775), Perciformes, Priacanthidae, moontail bullseye.

**Genus** ***Lecithostaphylus*** **Odhner, 1911**

ZooBank: urn:lsid:zoobank.org:act:431229B4-A8F9-4107-B3A4-779055CD30F4.

***Lecithostaphylus nitens*** (**Linton, 1898**) **Linton, 1940**

ZooBank: urn:lsid:zoobank.org:act:EDDA9CF7-19AA-4386-AAE2-BEBF6ABC566D.

Record from off New Caledonia: Bray & Justine (2008a).

New Caledonian host: *Tylosurus crocodilus* (Péron & Lesueur, 1821), Beloniformes, Belonidae, hound needlefish.

Discussion: This species is reported only from belonids of the genera *Tylosurus*, *Platybelone* and *Ablennes*, mostly from the former. It was originally reported in ‘*Tylosurus caribbaeus*’ [? *T. acus*] from off Woods Hole, Massachusetts in the Northwestern Atlantic Ocean. Most reports are from the northwestern Atlantic or Gulf of Mexico, including one from *T. crocodilus* (see Overstreet, 1969). Machida & Kuramochi (2000) reported this species from the Pacific Ocean off Japan and the Philippines.

**Genus** ***Overstreetia*** **Bray 1985**

ZooBank: urn:lsid:zoobank.org:act:DE25FCA5-450F-448E-9523-78F12BDB65A5.

***Overstreetia cribbi*** **sp. n.**

ZooBank: urn:lsid:zoobank.org:act:2047A8D5-A5AE-49E9-B521-7EF17CA9F9EA.

Host: *Atherinomorus lacunosus* (Forster, 1801), Atheriniformes, Atherinidae, hardyhead silverside.

Site: digestive tract.

Locality: Anse Vata, Nouméa (22°18′30″S, 166°25′50″E, 03/10/2008).

Specimens: Holotype MNHN JNC2656, Paratype BMNH 2014.1.31.1.

Etymology: This species is named after our colleague Dr. Tom Cribb, of the University of Queensland, who has contributed immeasurably to our understanding of the taxonomy and biology of digeneans.

**Figure 6:**
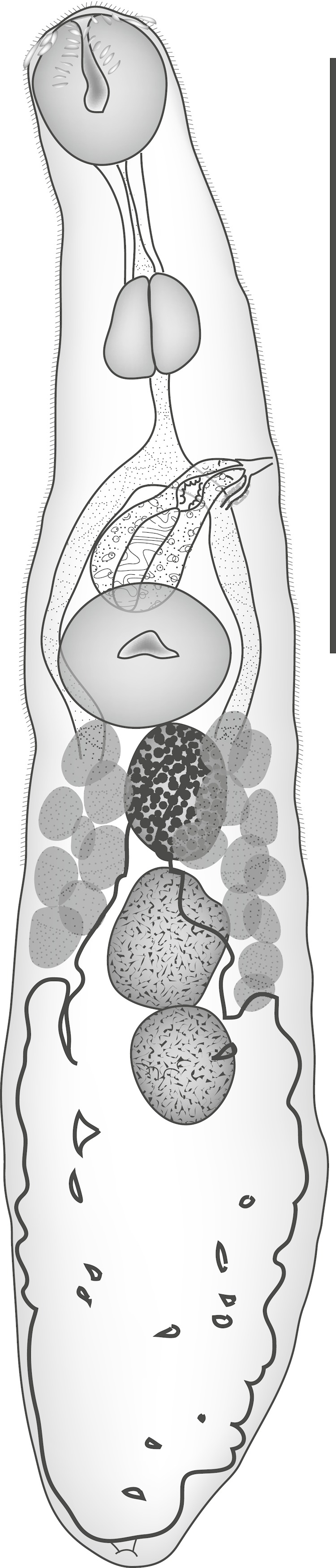
*Overstreetia cribbi* n. sp. *Overstreetia cribbi* n. sp., ventral view of holotype, uterus in outline. Scale bar 500 µm.

**Figure 7:**
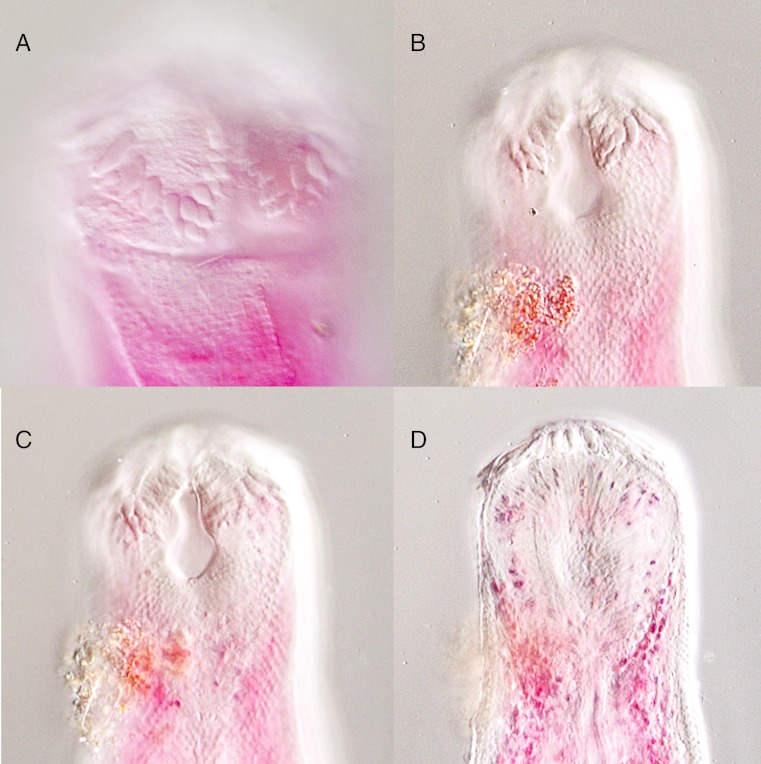
*Overstreetia cribbi* n. sp. *Overstreetia cribbi* n. sp., photographs. (A) Anterior end of paratype showing spination. (B) Anterior end of holotype showing spination in ventral plane. (C) Anterior end of holotype showing spination in plane slightly dorsal to figure 7B, also showing ventral aperture of oral sucker. (D) Anterior end of holotype showing spination in dorsal plane.

Based on 2 ovigerous specimens. Measurements on Table 3. Body elongate, narrow fusiform (Fig. 6). Tegument spinous posteriorly as far as anterior hindbody. Enlarged circum-oral spines present around oral sucker and reaching posteriorly forming arc alongside aperture (Fig. 7), up to 36 long. Oral sucker subglobular, with narrow ventro-terminal aperture. Ventral sucker circular, in anterior half of body, slightly wider than oral sucker. Forebody long. Prepharynx long thick-walled. Pharynx oval. Oesophagus shorter than prepharynx. Intestinal bifurcation in posterior forebody. Caeca pass into uterine area, terminations obscured by eggs.

**Table 3:** Original measurements of *Overstreetia* spp. Bold figures indicate distinctions from *O. cribbi*.

Species	*O. cribbi n. sp.*		*O. sodwanaensis*	*O. olsoni*
Host	*Atherinomorus lacunosus*		*Pranesus pinguis*	*Atherinomorus capricornensis*
*n*	2		1	1
	Holotype	Paratype	Holotype	Paratype
Length	1,313	1,250	**1,947**	1,030
Width	255	237	**305**	134
Forebody length	478	420	736	421
Oral sucker length	163	142	136	78
Oral sucker width	115	104	116	73
Prepharynx length	59	34	173	103
Pharynx length	84	72	111	49
Pharynx width	90	73	98	50
Oesophagus length	57	102	210	116
Intestinal bifurcation to ventral sucker	110	63	64	66
Pre-vitelline distance	584	538	889	491
Long vitelline field	256	248	522	252
Short vitelline field	214	210	530	233
Ventral sucker length	121	155	191	67
Ventral sucker width	141	121	206	73
Cirrus-sac length	258	?	274	142
Cirrus-sac width	57	?	83	52
Ventral sucker to ovary distance	0	0	21	4
Ovary length	114	98	136	72
Ovary width	94	82	77	64
Ovary to anterior testis distance	0	0	93	48
Anterior testis length	101	92	156	75
Anterior testis width	84	73	184	57
Distance between testes	0	0	0	0
Posterior testis length	97	101	173	69
Posterior testis width	87	90	153	53
Post-testicular distance	378	404	461	274
Post-vitelline distance	472	458	496	297
Post-uterine distance	13	33	64	61
Post caecal distance	?	?	178	153
Egg length	35–40 (38)	31–39 (36)	35–40 (37)	33–42 (38)
Egg width	14–23 (20)	18–24 (21)	19–26 (23)	17–24 (20)
Width %^*^	19.4	18.9	**15.6**	**13.0**
Forebody %^*^	36.4	33.6	37.8	**40.9**
Sucker length ratio	0.74	1.09	**1.41**	0.86
Sucker width ratio	1.23	1.17	**1.78**	1.00
Pharynx: oral sucker width ratio	0.78	0.70	0.85	0.68
Oral sucker length %^*^	12.4	11.4	**6.96**	**7.55**
Pharynx length %^*^	6.36	5.73	5.68	4.73
Ventral sucker length %^*^	9.20	12.4	9.82	**6.53**
Oesophagus length %^*^	4.32	8.20	**10.8**	**11.3**
Pre-vitelline distance %^*^	44.5	43.0	45.6	47.6
Long vitelline field %^*^	19.5	19.8	**26.8**	**24.5**
Ovary length %^*^	8.69	7.81	6.98	6.97
Ovary to anterior testis distance %^*^	0	0	**4.76**	**4.69**
Anterior testis length %^*^	7.70	7.37	8.02	7.25
Posterior testis length %^*^	7.39	8.09	8.88	6.67
Post-testicular distance %^*^	28.8	32.4	**23.7**	**26.6**
Post-vitelline distance %^*^	35.9	36.6	**25.5**	**28.8**
Post-uterine distance %^*^	0.96	2.61	**3.31**	**5.91**
Postcaecal distance %^*^	?	?	4.57	16.0
Cirrus-sac length %^*^	19.7	?	**14.1**	**13.7**
Intestinal bifurcation to ventral sucker %^**^	23.1	15.1	**8.71**	15.6

**Notes.**

* % of body-length.

** % of forebody.

Testes 2, oval, entire to slightly irregular, tandem, contiguous, in anterior half of hindbody. Cirrus-sac broadly claviform, posterior end overlaps anterior edge of ventral sucker. Seminal vesicle allantoid, undivided, surrounded by gland-cells, in proximal region of cirrus-sac. Pars prostatica vesicular. Ejaculatory duct short, thick-walled. Genital atrium small. Genital pore submarginal, sinistral, bifurcal.

Ovary oval, entire, contiguous with ventral sucker and close to anterior testis. Proximal female system obscured by eggs. Uterus runs posteriorly from ovary passes ventrally over testes, fills bulk of body posterior to anterior testis, presumably reaches extracaecally, but caeca obscured by eggs. Metraterm short, muscular, with narrow sheath of gland-cells. Eggs numerous, operculate, tanned. Vitellarium forms 2 lateral fields of few (8–9 aporal, 11 poral) irregularly oval follicles between levels of ventral sucker and posterior testis.

Excretory pore terminal. Excretory vesicle anterior extent and shape obscured by eggs.

## Discussion

According to the key in Bray (2008) only members of two zoogonid genera have tandem testes, namely *Overstreetia* and *Pseudopalaeorchis* Kamegai, 1970. Since the key was produced two further zoogonid genera have been described with tandem testes, *Whitegonimus* Jeżewski, Zdzitowiecki & Laskowski, 2009 and *Oesophagotrema* Chaari, Derbel & Neifar, 2011 (Jeżewski, Zdzitowiecki & Laskowski, 2009; Chaari, Derbel & Neifar, 2011). Only *Overstreetia* is known to exhibit enlarged circum-oral spines.

Only six ovigerous specimens of *Overstreetia* spp. have been reported. The genus was erected based on two specimens of the type species *O. sodwanaensis* Bray, 1985 from *Pranesus pinguis* (Lacepède, 1803) off Sodwana, Natal, South Africa (Bray, 1985). Subsequently, two ovigerous specimens and one immature specimen of *O. olsoni* Bray & Cribb, 2006 were described from the Capricorn silverside *Atherinomorus capricornensis* (Woodland, 1961) off Heron Island, Queensland, Australia (Bray & Cribb, 2006). Now we have discovered two ovigerous specimens from *Atherinomorus lacunosus* off New Caledonia. We have re-measured the holotype of *O. sodwanaensis* (BMNH 1983.8.3.1) and the paratype of *O. olsoni* (BMNH 2005.3.11.6) and included the data in Table 3.

**Figure 8:**
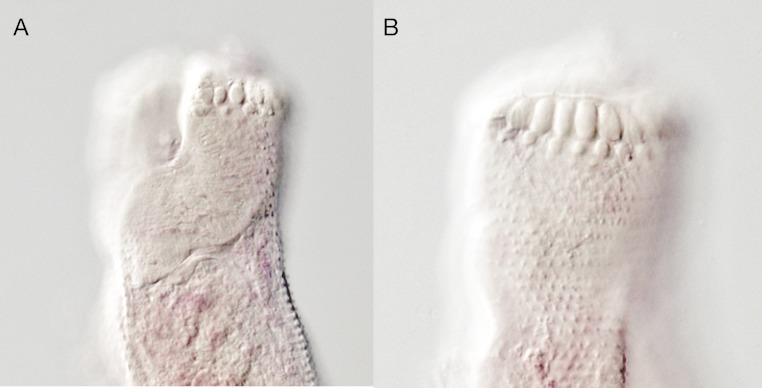
*Overstreetia sodwanaensis.* *Overstreetia sodwanaensis* Bray, 1985, photographs. (A) Anterior end of holotype (BMNH 1983.8.3.1) showing spination in ventrolateral plane, also showing ventral aperture of oral sucker. (B) Anterior end of holotype showing spination in dorsolateral plane.

**Figure 9:**
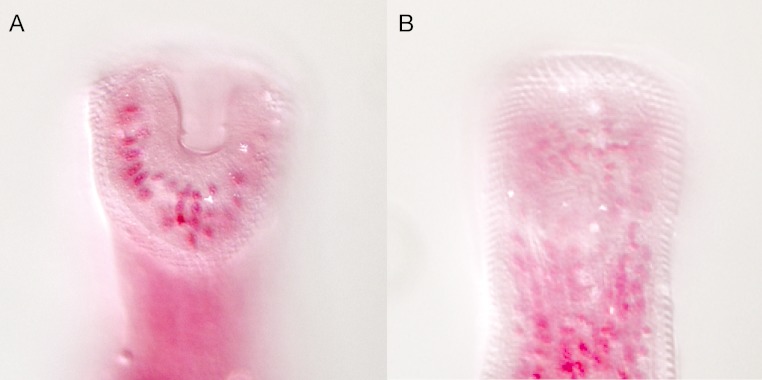
*Overstreetia olsoni.* *Overstreetia olsoni* Bray & Cribb, 2006, photographs. (A) Anterior end of paratype (BMNH 2005.3.11.6) showing spination in ventral plane, also showing ventral aperture of oral sucker. (B) Anterior end of paratype showing spination in dorsal plane.

These two specimens appear to represent a new species. They differ from the described species most obviously in the oral spination. In *O. cribbi* the enlarged spines form an arc beside the aperture of the oral sucker and pass dorsally around the oral sucker region. This contrasts with the condition in *O. sodwanaensis* where the oral spine rows are limited to the anterior part of the oral sucker region (Fig. 8), and the condition in *O. olsoni* where there are no noticeably enlarged circum-oral spines (Fig. 9).

In *O. sodwanaensis* the cirrus-sac is recurved, with a long internal seminal vesicle. The body is narrower, the ventral sucker is relatively larger, the oesophagus, vitelline field and ovary to anterior testis distances are relatively greater and the oral sucker size, post-testicular distance and post-vitelline distance are relatively smaller (Table 3).

In *O. olsoni* the body is narrower, the forebody, oesophagus, vitelline field and ovary to anterior testis distances are relatively greater and the oral and ventral sucker sizes, post-testicular distance and post-vitelline distance are relatively smaller (Table 3).

In our discussion of the hosts of *Overstreetia* above we have used the names given in the original papers, but recently Kimura et al. (2007) have reviewed and restudied Indo-Pacific *Atherinomorus* [syn: *Pranesus*] spp. and synonymised *A. capricornensis* with *A. lacunosus* and recognised *A. pinguis*. Presuming that the host identifications are correct, then the *Overstreetia* from the Great Barrier Reef and New Caledonia are from the same host species, but differ distinctly. The host of the South African species may also be conspecific, as Kimura et al. (2007) considered some of the subspecies of *A. pinguis* as well as some specimens referred to *A. pinguis* by various authors to be synonymous with *A. lacunosus.*

**Genus** ***Sacculoacetabulum*** **Machida & Kuramochi, 1999**

ZooBank: urn:lsid:zoobank.org:act:9115FED3-87BC-44C7-A310-8C9638F18C98.

***Sacculoacetabulum ohjibah*** **Machida & Kuramochi, 1999**

ZooBank: urn:lsid:zoobank.org:act:37EC1F47-C1B4-43C2-96F4-223DC92CCB0F.

Record from off New Caledonia: Bray, Cribb & Justine (2010).

New Caledonian host: *Triodon macropterus*.

Discussion: This species was originally reported in this host, from off Okinawa, Japan (Machida & Kuramochi, 1999). Our record from off New Caledonia is the only other report of this species.

### Zoogonidae immature

Host: *Parupeneus multifasciatus* (Quoy & Gaimard, 1825), Perciformes, Mullidae, manybar goatfish.

Site: digestive tract.

Locality: Off Récif Kué, Middle of Reef (22°36′30″S, 166°32′E, 09/12/2008).

Specimens: JNC2827.

### Zoogeography

The zoogonid fauna of New Caledonian waters as described here is small, and probably represent a low proportion of the complete fauna as the Zoogonidae (and particularly the Lepidophyllinae) is one of the relatively few digenean families with a good representation in the deep-sea, i.e., off the continental shelf (Bray, 2004). Nevertheless, we will list the distribution of the 13 species which have been reported from New Caledonian waters, bearing in mind that these results are preliminary.

Four species (31% of the fauna) are endemic: *Deretrema combesae*, *Deretrema combesorum*, *Dupliciporia lanterna* and *Overstreetia cribbi* n. sp.

One species (8%) is restricted to South Western Pacific close to New Caledonia (FAO Major Fishing Area 71): *Diphterostomum tropicum*.

Four species (31%) are found in the northern and southern Western Pacific (FAO 61 & 71): *Diphterostomum plectorhynchi*, *Parvipyrum acanthuri*, *Deretrema triodontis* and *Sacculoacetabulum ohjibah*.

One species (8%) is reported in the Western and Central Pacific (FAO 61, 71 & 77): *Deretrema acutum*.

One species is reported from sites across the Pacific Ocean (FAO 61, 71 and 87): *Zoogonus pagrosomi*.

Two species (15%) are cosmopolitan (FAO 21, 27, 31, 37, 57, 61, 67 & 71): *Zoogonoides viviparus*, *Lecithostaphylus nitens*. These are probably cryptic complexes.

## Additional Information and Declarations

### New Species Registration

The following information was supplied regarding the registration of a newly described species: The new species is entered in ZooBank and its LSID: urn:lsid:zoobank.org:act:FC32CC29-F6DD-46DC-B2F2-84F3953B2991.
